# Suicide Risk Factors among Polish Adults Aged 65 or Older in 2000–2018 Compared with Selected Countries Worldwide

**DOI:** 10.3390/ijerph18189921

**Published:** 2021-09-21

**Authors:** Witold Śmigielski, Karolina Małek, Tomasz Jurczyk, Karol Korczak, Robert Gajda, Alicja Cicha-Mikołajczyk, Jerzy Piwoński, Joanna Śmigielska-Kolańska, Janusz Śmigielski, Wojciech Drygas, Piotr Gałecki

**Affiliations:** 1 Department of Demography, University of Lodz, 41, Rewolucji 1905 St., 90-214 Lodz, Poland; 2 Department of Epidemiology Cardiovascular Disease Prevention and Health Promotion, The Cardinal Stefan Wyszyński National Institute of Cardiology, 42 Alpejska St., 04-628 Warsaw, Poland; acicha@ikard.pl (A.C.-M.); jpiwonski@ikard.pl (J.P.); wdrygas@ikard.pl (W.D.); 3 Faculty of Psychology, Warsaw University, 26/28 Krakowskie Przedmieście St., 00-927 Warsaw, Poland; kmalek@psych.uw.edu.pl; 4 The Specialist Family Clinic of Bemowo District in the Capital City of Warsaw, Gen. T. Pełczyńskiego 28 E. St., 01-471 Warsaw, Poland; tj.jurczyk@gmail.com; 5 Department of Computer Science in Economics, University of Lodz, 41 Rewolucji 1905 St., 90-214 Lodz, Poland; karol.korczak@uni.lodz.pl; 6 Center for Sports Cardiology at the Gajda-Med Medical Center in Pułtusk, Piotra Skargi 23/29 St., 06-100 Pułtusk, Poland; gajda@gajdamed.pl; 7 Specialist Psychiatric Health Center in Lodz, Babiński Hospital, 159 Aleksandrowska St., 91-229 Lodz, Poland; Joanna.kolanska@hotmail.com; 8 Department of Health Sciences, State University of Applied Sciences in Konin, 1 Przyjaźni St., 62-510 Konin, Poland; j.smigielski@neostrada.pl; 9 Department of Preventive and Social Medicine, Medical University of Lodz, 4 Tadeusza Kościuszki St., 90-419 Lodz, Poland; 10 Department of Adult Psychiatry, Medical University of Lodz, 4 Tadeusza Kościuszki St., 90-419 Lodz, Poland; piotr.galecki@umed.lodz.pl

**Keywords:** suicide, people aged 65 or older, Poland, educational level, marital status, mortality, death risk

## Abstract

The aim of this study was to determine the tendencies of change in suicide frequency among Polish adults aged 65 or older, recognize the importance of available socio-demographic data (age, sex, marital status, and education attainment level) and provide an in-depth psychological understanding of the obtained results. We analysed the influence of education and marital status on suicide risk in the Polish adult population aged 65 or older, which has not been previously presented in publications related to the Central Statistical Office or any other research. Our results indicated that male adults aged 65 or older that were single or divorced and with a lower education had a higher risk of death by suicide. In female adults aged 65 or older, those with higher education and who were divorced or married had a higher risk of fatal suicide behaviour meanwhile, single women and widows had a lower risk. The dominant method of suicide among Polish older adults was suicide by hanging, regardless of sex; female older adults were more likely to die by suicide by poisoning or jumping from a height, and male older adults were more likely to die by shooting with a firearm. Although data from recent years highlights a downward trend for suicide rates in Polish older adults, the problem cannot be considered solved.

## 1. Introduction

A report from the World Health Organization (WHO) on suicide prevention states that 800,000 suicides occur yearly, and it appears that the number of deaths due to suicide attempts is now greater than those due to armed conflict, murder or natural disasters [1]. Suicide is defined as the act of intentionally terminating one’s own life [2,3]. It is a multidisciplinary phenomenon that is studied and described in the fields of medicine, public health, psychology, law, sociology, philosophy and culture [4,5]. 

According to the WHO, there are indications that for each adult who dies of suicide there may be more than 20 others who have attempted suicide. Data on suicide attempts that result in death indicate that the risk of suicide increases with age; indeed, suicide rates are highest in persons aged 70 years or over for both men and women in almost all regions of the world [1]. In Poland, as per our results, the highest number of suicides occurred in the 50–59 age group; although a significant number of people over 70 years of age choose suicide, this problem is not sufficiently publicised [6].

Data from the European Commission from 2011 to 2015 indicates that suicides constituted about 1.1% of the total number of deaths in the European Union (EU; Eurostat database). This value is both large and small; it could be considered small because 1% does not seem to be a big number. However, if the dramatic circumstances surrounding the decision to attempt suicide are taken into consideration, this 1% becomes an alarming value—one that European society cannot accept, especially if one realises that 50,000 EU citizens die this way every year. Poland is characterised by a rather low suicide indicator (male: 26.2/100,000; female: 4.1/100,000 adults aged 65 or older). Notably, for most countries in Europe the rates for death caused by suicide significantly declined from 2000 to 2017 by an average of about 30% for males and 40% for females (see Supplementary Table S1).

The literature reports different risk factors related to suicidal death in adults aged 65 or older. It has been proved that somatic conditions (e.g., neurobiological factors) [7], chronic and/or lethal diseases such as Parkinson’s disease [8], epilepsy [9,10] Huntington’s disease [11], AIDS, cancer, cardiovascular diseases [12], socio-economic factors (e.g., unemployment, challenging economic situation) [13], lack of social support [14] and family history [15] increase the risk of suicidal death. Psychological factors and mental health conditions are also recognized as significant. These include psychopathological factors, mental disease/disorder [16,17,18], personality disorder traits [19], personality traits such as hopelessness, neuroticism, extroversion [20], emotion regulation [21], thwarted belongingness and perceived burdensomeness [19]. The research shows that approximately 90 percent of suicide cases meet the criteria for a psychiatric disorder [3]. Additionally, one meta-analysis showed that the most unique and universal factor contributing to suicide is psychological crisis accompanied by adaptation problems and mental disorder [17].

Several risk factors specific to people aged 65 or older can also be identified, for example, some research shows that people aged 65 or older suicide attempters have higher levels of orderliness—a conscientiousness subcomponent—than non-suicidal depressed individuals, which represents a difference in comparison to younger people making their first suicide attempt who are more frequently characterised by high neuroticism, low extraversion, and anti-social and borderline PD traits [22]. Another paper points to psychiatric and neurocognitive disorders, bereavement, cognitive impairment, decision making and cognitive inhibition, social exclusion, physical illnesses and physical and psychological pain as the main risk factors for adults aged 65 or older [23].

Other more transient factors that reflect an imminent risk of suicide crisis include unbearable mental pain and related experiences of depression and hopelessness, which may lead to a suicidal state of mind. This is understood as an experience where suicide itself becomes the only force that can balance tension, suffering and hopelessness, and may result in suicide attempts [24]. This aligns with research results showing that the strongest risk factor for fatal suicide behaviour is the previous occurrence of a suicide attempt [25].

The results of empirical research that highlight the multiple aforementioned risk factors for suicidal death provide a better understanding of the complexity and multi-causality of suicidal behaviour.

In most countries, the frequency of fatal suicide behaviour attempts is the highest among people aged 65 or older [26]. Additionally, although the number of suicide attempts (non-fatal suicides behaviours) decreases with age, along with a decrease in psychiatric problems, legal and financial stressors and relationship problems, the number of fatal suicides behaviours still increases, indicating greater determination and decisiveness in the people undertaking such behaviours [27].

Accordingly, herein, we discuss a specific group of adults aged 65 or older who died from suicide in Poland; namely, we do not address the entire suicidal area, and suicidal ideation and attempts are not the focus of this work. The problem of suicide among adults aged 65 or older has become an important public health issue as previous research shows that the suicide rates for men and women in Europe are highest among people aged 65 or older in comparison to other age groups, with higher rates for men in all older age groups (male–female rate ratio r = 4.0) [28].

The general aim of this study was to determine the change tendencies in suicide frequency among Polish adults aged 65 or older. The specific aim of our research was to recognize the importance of available socio-demographic data (age, sex, marital status, education attainment level) and provide an in-depth psychological understanding of the obtained results. Among the socio-demographic factors, we decided to focus on sex, age group, civil status and education level. Additionally, as the research shows, marital status is an important socio-demographic factor associated with the suicide ratio in adults aged 65 or older [29,30].

Our analysis was based on official state statistical data from the last 20 years, which is an important advantage of this study; the analysed data are reliable and characterize the whole Polish population. However, we had no access to other interesting factors that are not collected by the Polish Central Statistical Office. It has to be underlined that this study only analyses sociodemographic risk factors in those who have died by suicide and not in the broad spectrum of suicidal behaviour.

In connection with the abovementioned aims, we established the following research questions:

1. Is the frequency of fatal suicide behaviour attempts among Polish adults aged 65 or older decreasing, increasing or stable?
2. Does sex, age group, civil status or education level differentiate, in a statistically significant way, the risk of a fatal suicide behaviour attempt among Polish adults aged 65 or older?
3. Could psychological theory and research help to explain the observed statistical relationships?

## 2. Materials and Methods

We analysed Central Statistical Office (CSO) data, including detailed information that is not usually published in official reports (e.g., education, marital status, and the exact cause of death by suicide according to the classification set forth by the International Classification of Diseases, Tenth Revision). In analysing marital status, single individuals were understood as those who had never been married, married people were those who were still in a legal marriage, widowed individuals were those whose spouse died during their marriage, and divorced individuals were those whose marriage was dissolved by a court decision. If a widowed or a divorced person remarried, and this union was not dissolved, they were regarded as a married person. The abovementioned data were obtained from the National Institute of Cardiology status project no. 2.17/I/19. We also obtained the relevant agreement from the Bioethical Commission of the National Institute of Cardiology. We limited our data analysis to adults aged 65 or older (counted annually) who died in the period from 2000 to 2018. This allowed for a more comprehensive analysis of the issued and the identification of the dependencies that characterise them. Our analysis was based on 3417 men aged 65 or older and 931 women aged 65 or older (*n* = 4348) who died due to suicide in Poland in the years 2000, 2005, 2010, 2015 and 2018 in comparison to all people aged 65 or older in Poland who died in the abovementioned period of time (males: 637,198; females: 760,898; total: 1,398,096).

The analysis of raw data was then augmented by the determination of death rates owing to suicide (per 100,000 inhabitants); specifically, we measured the impact of socio-demographic variables (e.g., education and marital status) by comparing the structure of the abovementioned traits among adults aged 65 or older who died by suicide with the structure of the abovementioned traits among the total number of adults aged 65 or older who died in the same period of time. We used the relative risk (RR) for these comparisons together with the determination of their *p*-value; statistical significance was set at *p* = 0.05. Relative risk is the ratio of the probability of an event occurring in the exposed group versus the probability of the event occurring in the nonexposed group. We used MEDCALC statistical software and Excel for statistical analysis. 

## 3. Results

Data on suicide rate changes among adults aged 65 or older in Poland from 2000 to 2018 are presented in Table 1. The total number of suicides among male adults aged 65 or older in Poland at the beginning of the 21st century was characterised by an upward trend; in 2015, it was nearly 30% higher than in 2000 (i.e., from 578 to 745), with the highest dynamic growth recorded among men aged ≥85 years old. In 2018, compared with 2015, there was a decrease in the total number of deaths by suicide in all age subgroups among male adults aged 65 or older (75–84 years: a decrease from 216 to 170; 85+ years: from 82 to 77). An increasing trend was seen for the youngest group of adults aged 65 or older (i.e., 65–74 years: an increase from 447 to 462). 

In the first decade of the 21st century, the number of suicides among female adults aged 65 or older showed a slightly decreasing tendency (in 2000, there were 202 deaths by suicide, compared to 196 in 2010); in the following years, there were further and more significant reductions (i.e., 2015: 164 cases; 2018: 169 cases). 

**Figure 1:**
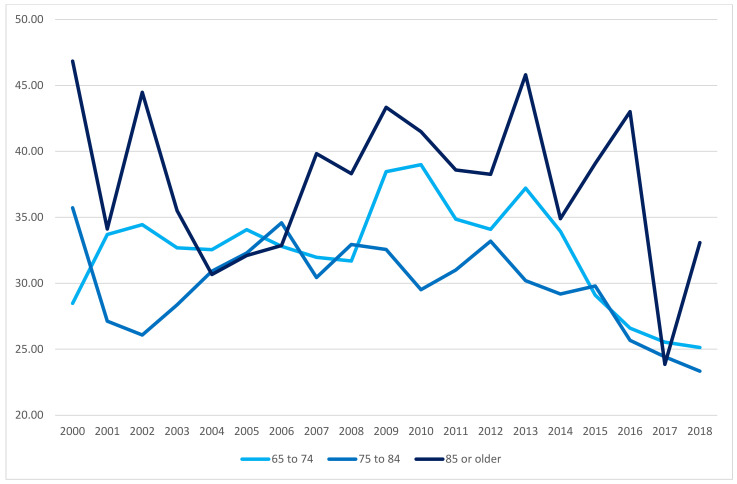
Suicide rates (per 100,000 population) among male adults aged 65 or older by age group from 2000 to 2018. Source: Polish Central Statistical Office (CSO).

**Figure 2:**
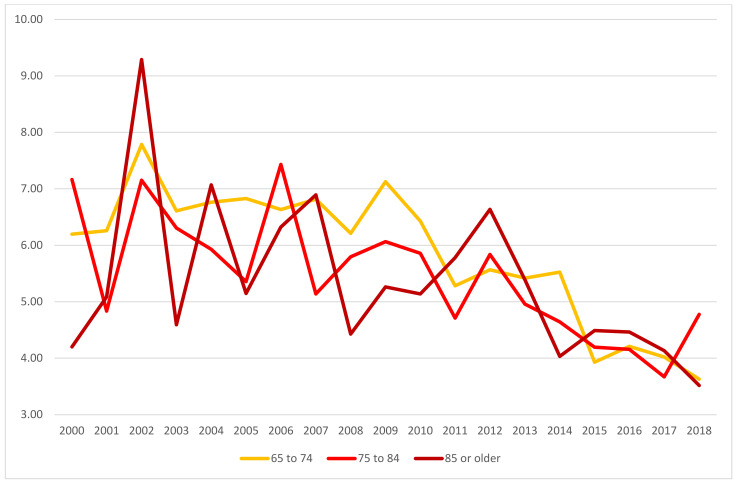
Suicide rates (per 100,000 population) among female adults aged 65 or older by age groups from 2000 to 2018. Source: Polish Central Statistical Office (CSO).

In the first decade of the 21st century, the suicide rates per 100,000 inhabitants among male adults aged 65 or older (considering age structure changes) slightly increased, and a significant decrease was observed in the following years (Figure 1, Table 1). Among female adults aged 65 or older, these rates showed a constant decreasing tendency (Figure 2, Table 1). 

In 2018, the highest suicide rate by sex was observed in male adults aged over 85 years (34.9/100,000) and female adults aged 75–84 (4.9/100,000). Moreover, in the first decade of the 21st century, the incidence of fatal suicides behaviours among total deaths in male adults aged 65 or older showed an increasing tendency (2000: 4.89/1000; 2005: 5.54/1000; 2010: 6.00/1000), although the subsequent years showed a decrease—in 2018, the value was almost equal to that observed in 2000 (2018: 4.87/1000). The incidence of fatal suicides behaviours among total deaths in female adults aged 65 or older was characterized by a steadily decreasing trend (2000: 1.44/1000; 2010: 1.34/1000; 2018: 0.98/1000; Table 1).

Considering education, male adults aged 65 or older with secondary education or higher were characterised by a lower suicide risk (RR: 0.59 and 0.75, respectively), while those with primary education were characterised by a higher risk (RR = 1.10). Moreover, we noted a significantly higher risk of fatal suicide behaviour in divorced (RR = 1.67) and single male adults aged 65 or older (RR = 1.62); those that were married showed a lower risk (RR = 0.86). Among female adults aged 65 or older, we observed a higher risk of fatal suicide behaviour in those with higher education (RR = 1.49) and basic vocational education (RR = 1.36). Moreover, divorced female adults aged 65 or older were also characterised by a higher risk of fatal suicide behaviour (RR = 1.56); however, unlike male adults aged 65 or older, married female adults aged 65 or older showed a higher risk of death due to suicide (RR = 1.47; Table 2).

**Figure 3:**
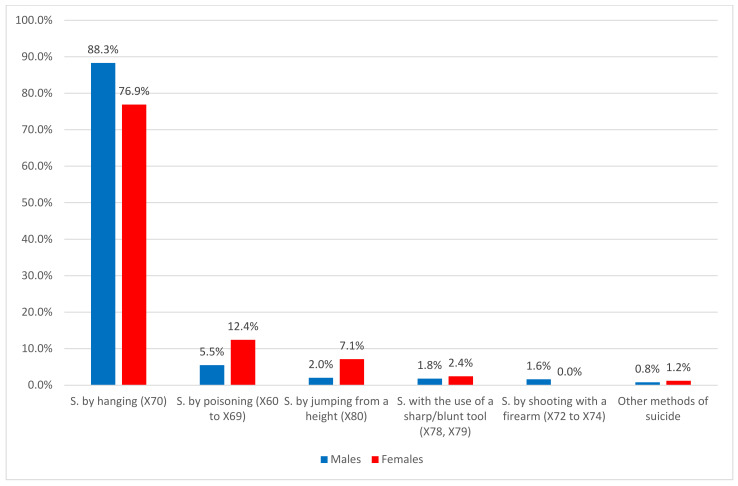
Suicide methods among adults aged 65 or older in Poland by sex, 2018. Source: Author’s own calculations based on data from the Polish Central Statistical Office (GUS); S. = Suicide.

We also analysed suicide methods, and observed that suicide by hanging was the most common method in both male and female adults aged 65 or older (2018, men: 88%; women: 77%). However, female adults aged 65 or older died by suicide by deliberate poisoning (12.4% and 5.5% for females and males, respectively) and jumping from a great height (7.1% and 2.0% females and males, respectively) more often than their male counterparts. Meanwhile, male adults aged 65 or older died by suicide by deliberate shooting with a firearm more often than their female counterparts (1.6% and 0% for males and females, respectively) (see Table 3, Figure 3).

## 4. Discussion

The results obtained in this study indicate the areas for further research and call for an effort to explain and understand the phenomena underlying suicidal death among adults aged 65 or older in Poland.

There are many theories that aim to explain suicide as a complex and multidisciplinary problem [5]. One perspective to consider in regard to adults aged 65 or older suicide is these individuals’ psychosocial development [31]. In the theory developed by Erikson, human development continuously advances through a number of stages and individuals must face the psychological “crisis” that characterises each of these stages; namely, individuals are challenged by certain developmental tasks. According to Erikson [32] and researchers following his work [31,33], the final stage of human development is focused on integrity vs. despair. It is a time of developmental crisis in which individuals have to reflect upon their life and balance it so that they can maintain integrity and satisfaction, regardless of declining health and the looming prospect of death. Accordingly, failure to deal with this existential task can lead to a lack of purpose and meaning, contribute to mental distress and potentially facilitate suicide. From this perspective, it may be easier to understand why the numbers for death by suicide in this group are generally higher.

Our results on the topic indicate that men with lower education are at greater risk of suicide, which is in agreement with other studies. As an example, a large study in Italian adults aged 65 or older conducted from 1993 to 2010 showed that education was negatively correlated with suicide rates [34]. Thus, education might be a mediating factor related to lower socioeconomic status, such as unemployment [35] or addiction (e.g., alcohol addiction) [36]. It has been proven that both factors are associated with higher suicide rates [37,38,39].

Our study also revealed important sex differences in Polish adults aged 65 or older; higher education in females was associated with higher suicide rates. In an American study by Stack [40] on African Americans, higher education and higher intelligence correlated with higher mental disorder scores and higher relative risk of suicide in situations where there was a significant mismatch between these factors and the expected and anticipated socio-economic benefits (i.e., better jobs, higher income and better housing) [40,41]. These results may, to some extent, explain the situation of highly educated female adults aged 65 or older in Poland if we consider the historical and social group context in the country. For women, achieving a higher education in post-war Poland came with many different challenges, and meeting these challenges required investment and effort. These women’s hopes for a better quality of life, which might have been the reason for such efforts, may not have been fulfilled. In contrast, the relative risk of suicide was lower for female adults aged 65 or older with lower levels of education. 

Prior literature discusses the nonlinear relationships between education and suicide risk in adults aged 65 or older, and shows a correlation with both low and high education [42]. Thus, our results provide new conclusions that underline the significance of sex-related factors in Polish adults aged 65 or older. Another result illustrating the existence of sex differences in suicidal adults aged 65 or older relates to our results regarding marital status.

Our results indicate that higher suicide rates were connected to marital status. Most studies show a linear effect between marital status and relative risk of suicide, which can be explained by study methodology, for example, cursory questions in questionnaires (i.e., “Are you married or single?” [43]) or by analysing both sexes at the same time. In our data, married and divorced female adults aged 65 or older died by suicide at higher rates than did the general population. Meanwhile, male adults aged 65 or older who were divorced and had a bachelor’s degree died by suicide more often than the general population. Thus, there is a potential underlying socio-psychological context for higher suicide risk in adults aged 65 or older. For this age group, regardless of gender, divorce is mostly experienced as a failure and a reason to be ashamed; data from CBOS in 1991 show that only 15% of Polish people considered divorce an acceptable termination of marriage [44]. This result is consistent with the previous research indicating that the suicide risk in divorced individuals is higher than for non-married individuals in both men and women [45].

This means that not only is it probable that these divorced adults aged 65 or older are more frequently alone, but also that they may see their situation as a powerful stressor. Similar results concerning divorce were found in studies conducted in Ireland, which has similarities with Poland [29]. The results of our study show that males with higher educational attainment had a higher relative risk of suicidal death. This result is consistent with other studies, indicating that individuals with higher educational achievement may be more prone to suicide risk when facing failures, public shame and high premorbid functioning [46].

Moreover, based on our results, we hypothesize that the quality of the marriage of both male and female adults aged 65 or older may be a crucial factor for their quality of life and their suicide risk. Some studies show that low relationship satisfaction is an important risk factor in adults aged 65 or older —indeed, more important than being alone [29]. Our study confirms these results for the female sample, where we found a higher relative risk for divorced and married women. Additionally, research shows that in couples, marital satisfaction is associated with health and well-being over time [37]. This might be especially significant considering that women aged 65 or older frequently end up providing care for their male partners. Studies show that the mean life expectancy for women is 81.8, while for men it is 74.1 [47]. Another study revealed that male adults aged 65 or older have higher morbidity and mortality than female adults aged 65 or older, considering the ratios for oncological, cardiovascular and neurological diseases [48]. The results showing a higher suicide rate for adult married women aged 65 or older seem to support this conclusion.

Another part of the analysis conducted within our study focused on the choice of suicide method in adults aged 65 or older. A previous study [49] concluded that acceptability, availability and lethality are important factors affecting the choice of suicide method and should be considered when developing suicide prevention programs. Our results regarding suicide methods showed that hanging was the most common suicide method among Polish adults aged 65 or older; in fact, previous research suggests that hanging is viewed as accessible and it is perceived as fast, “clean”, and not very painful [50]. Empirical results also indicate that adults aged 65 or older who chose hanging were more likely to have mental illness, whereas this was the opposite for those using firearms. Another study supported this, showing that suicide by firearms was related to more reactive cases (i.e., a response to life events), as people who attempt suicide by this method often do not have a history of mental illness [49]. Meanwhile, another study found that violent methods of suicide were linked to lifelong impulsive-aggressive behaviour [51].

Moreover, based on our results, it seems that the percentage of people who use poisoning as a suicide method is rising; this may be due to the growing availability of psychiatric drugs accompanied by insufficient supervision and a lack of systematic care from psychiatric clinicians in Poland. Corroborating this, one study showed that there are approximately 90 psychiatrists per 1 million people in Poland, and most of them are in large population centres [52]. Furthermore, another study showed that the choice of drug poisoning as a suicide method is associated with the presence of physical/mental illness [53]. In another study, most self-poisonings by adults aged 65 or older were intentional, and opioids were the most frequent drugs used in fatal cases [54]. Another study shows that prescription opioid and benzodiazepine misuse is associated with suicidal ideation in adults aged 65 or older [55]. This gives greater credibility to our hypothesis regarding the increase in suicides by poisoning as the availability of this method may be an important factor contributing to its choice, as stated previously [38]. Still, we should also consider the social acceptability of the suicide method, which includes a wide range of social discourse contexts. As an example, media-induced contagious effects are highly significant, and were shown to have a stronger impact among youths and adults aged 65 or older compared to middle-aged adults [41].

Considering the international situation, it is worth highlighting that in general the frequency of fatal suicide behaviour deaths among adults aged 65 or older in European countries is decreasing. Thus, the tendency observed in Poland seems to be similar to that in other countries in the European region. In comparison to other Eastern European countries, the frequency of fatal suicide behaviour deaths among Polish adults aged 65 or older is one of the lowest (one exception is male adults aged 65–74, who had a suicide rate similar to those seen in other Eastern European countries). Aggregate statistics for European countries indicate that male adults aged 65 or older had a higher risk of fatal suicide behaviour death than female adults aged 65 or older. The risk of suicidal death is positively correlated with age in both sexes ([56], see also Table S1). 

From the perspective of suicide prevention, research into suicide methods seems to be particularly promising. It has been repeatedly shown that restricting access to the prevailing suicide method in a country decreases suicide rates, and that the lethality of the method used is significantly correlated with the degree of intention to die [57].

## 5. Limitations of the Study

We recognise that our study on suicides and their socio-demographic context among adults aged 65 or older in Poland has limitations. It is known that suicide itself is a multidimensional, multifactorial phenomenon and—due to our access to limited data provided by the Polish Central Statistical Office—this article only analyses some of the sociodemographic factors. Especially, we were not able to include other factors that are potentially of high importance for the issue in consideration (income level, specific life conditions, etc.). It should also be noted that this study only analyses sociodemographic risk factors in those who have died by suicide and not in the broad spectrum of suicidal behaviour.

## 6. Conclusions

Suicides in general, and specifically those in adults aged 65 or older are global problems. Considering the contemporary demographic changes that many countries are experiencing, which relate to the increase in adults aged 65 or older populations internationally, the challenges related to the analysed issues are highly likely to become increasingly significant. Although our study identified a dynamic increase in the number of suicides among adults aged 65 or older during the analysed years (mainly because of the increase in the number of suicides among males aged 65 or older, especially in the period 2000–2015), we need to note that this might also be explained by the increase in the size of the population of adults aged 65 or older. This is reinforced by the fact that the frequency of suicide attempts among male adults aged 65 or older in our sample remained more or less stable in the analysed years, although there was a drop in suicide rates among male adults aged 65 or older in the last period. Concomitantly, deaths by suicide in female adults aged 65 or older have dropped noticeably. Despite these decreases, we cannot say that the suicide problem has been solved in Poland. Rather, further in-depth research on suicide attempt risk factors and improvements in the tools available to deal with this multidisciplinary phenomenon are necessary in order to reduce these behaviours to the lowest possible level.

Generally, our results indicate the importance of early diagnosis and suicide prevention programs directed towards adults aged 65 or older, as it was found in previous research [58].

However, it is also important to acknowledge specific group characteristics, as our results yielded differences by marital status and sex. Specifically, accurate diagnostic procedures may be of great value for adults aged 65 or older; the diagnosis of mood disorders—one of the most important suicide risk factors—is often difficult because the symptoms may be hidden behind nonspecific complaints. Meanwhile, the typical complaints refer to somatic processes (either objective or experienced), and they might be underestimated and/or interpreted as simple signs of aging (e.g., insomnia, activity decrease, appetite loss). Moreover, depressive disorders may take the shape of dementia-like disorders, or even influence/increase existing dementia symptoms [59]. It is vital when planning prevention programs that the stakeholders (e.g., policy makers and healthcare professionals) devise interventions that consider the specific, empirically proven determinants of suicide for this age group, as well as problems associated with the availability, acceptability and lethality of suicide methods.

Our research provides more detailed information about specific socio-demographic determinants of suicidal death among adults aged 65 or older, and supports previously stated guidelines concerning the importance of precise diagnosis, the availability of help and building social awareness of these problems and opportunities for support [60]. We believe that it helps to improve early detection and intervention strategies. However, there is still much to be done.

## Figures and Tables

**Table 1:** Suicide rates among adults aged 65 or older in Poland from 2000 to 2018.

Data ^1^	Sex	Age Group	2000	2005	2010	2015	2018
Total number of cases	Males	65–74	363	427	452	447	462
		75–84	166	202	208	216	170
		85+	49	33	63	82	77
		65+	578	662	723	745	709
	Females	65–74	109	121	98	84	86
		75–84	80	63	77	57	61
		85+	13	16	21	23	22
		65+	202	200	196	164	169
Per 100,000 people	Males	65–74	29.6	35.0	39.7	30.6	26.4
		75–84	38.3	33.3	30.4	30.2	24.3
		85+	51.4	35.1	46.5	42.7	34.9
		65+	32.9	34.5	36.9	31.5	26.6
	Females	65–74	6.2	7.0	6.2	4.4	3.8
		75–84	9.3	5.3	6.1	4.4	4.9
		85+	4.9	6.2	5.5	4.5	3.9
		65+	7.0	6.3	6.1	4.4	4.2
Per 1000 deaths	Males	65–74	6.67	8.80	10.94	9.56	8.21
		75–84	3.86	3.95	3.89	4.17	3.40
		85+	2.36	1.66	2.45	2.37	1.95
		65+	4.89	5.54	6.00	5.59	4.87
	Females	65–74	2.98	4.05	3.85	3.03	2.58
		75–84	1.47	0.99	1.26	1.02	1.15
		85+	0.26	0.34	0.35	0.30	0.26
		65+	1.44	1.42	1.34	1.02	0.98

^1^ Source: Polish Central Statistical Office (CSO).

**Table 2:** Proportions and relative risks of female and male adults aged 65 or older who died due to suicide by education and marital status.

Sex	Demographic Factor	Category	2000, 2005, 2010, 2015, 2018 ^1^		RR	*p*-Value
			Suicide	Other Mortality Causes		
Male	Education level	Higher	152 (4.4%)	47,699 (7.5%)	0.59	<0.0001
		Secondary	425 (12.4%)	105,808 (16.6%)	0.75	<0.0001
		Vocational	924 (27.0%)	155,091 (24.3%)	1.11	0.0002
		Primary	1648 (48.2%)	279,448 (43.9%)	1.10	<0.0001
		Lower primary	193 (5.6%)	39,805 (6.2%)	0.90	0.1506
		Unknown	75 (2.2%)	9347 (1.5%)	1.50	0.0004
	Marital status	Single	273 (8.0%)	31,522 (4.9%)	1.62	<0.0001
		Married	1811 (53.0%)	390,489 (61.3%)	0.86	<0.0001
		Divorced	288 (8.4%)	32,176 (5.0%)	1.67	<0.0001
		Widowed	930 (27.2%)	162,715 (25.5%)	1.07	0.0231
		Unknown	115 (3.4%)	20,296 (3.2%)	1.06	0.5491
Female	Education level	Higher	48 (5.2%)	26,397 (3.5%)	1.49	0.0049
		Secondary	165 (17.7%)	120,856 (15.9%)	1.12	0.1210
		Vocational	115 (12.4%)	69,278 (9.1%)	1.36	0.0005
		Primary	496 (53.3%)	439,789 (57.8%)	0.92	0.0080
		Lower primary	93 (10.0%)	93,062 (12.2%)	0.82	0.0397
		Unknown	14 (1.5%)	11,516 (1.5%)	0.99	0.9806
	Marital status	Single	46 (4.9%)	47,462 (6.2%)	0.79	0.1051
		Married	232 (24.9%)	128,932 (16.9%)	1.47	<0.0001
		Divorced	72 (7.7%)	37,658 (4.9%)	1.56	<0.0001
		Widowed	558 (59.9%)	522,449 (68.7%)	0.87	<0.0001
		Unknown	23 (2.5%)	24,397 (3.2%)	0.77	0.2057

^1^ Source: Author’s own calculations based on data from the Polish Central Statistical Office (GUS).

**Table 3:** Suicide method in adults aged 65 or older by type and sex.

Suicide Type ^1^ (ICD-10 Code)	Males			Females		
	2000	2010	2018	2000	2010	2018
**Suicide by hanging (X70)**	526 (91.0%)	655 (90.6%)	626 (88.3%)	158 (78.2%)	164 (83.7%)	130 (76.9%)
**Suicide by fatal poisoning (X60-X69)**	6 (1.0%)	34 (4.7%)	39 (5.5%)	7 (3.5%)	16 (8.2%)	21 (12.4%)
**Suicide by jumping from a height (X80)**	18 (3.1%)	12 (1.7%)	14 (2.0%)	18 (8.9%)	6 (3.1%)	12 (7.1%)
**Suicide with the use of a sharp or blunt tool (X78-X79)**	12 (2.1%)	12 (1.7%)	13 (1.8%)	5 (2.5%)	4 (2.0%)	4 (2.4%)
**Suicide by shooting with a firearm (X72-X74)**	4 (0.7%)	6 (0.8%)	11 (1.6%)	0 (0%)	0 (0%)	0 (0%)
**Suicide by drowning (X71)**	6 (1.0%)	1 (0.1%)	2 (0.3%)	10 (5.0%)	2 (1.0%)	1 (0.6%)
**Other methods of suicide (X83-X84)**	2 (0.3%)	1 (0.1%)	2 (0.3%)	1 (0.5%)	2 (1.0%)	0 (0%)
**Charcoal-burning suicide** **(X76)**	2 (0.3%)	0 (0%)	1 (0.1%)	0 (0%)	1 (0.5%)	0 (0%)
**Suicide by truck (X81-X82)**	2 (0.3%)	2 (0.3%)	1 (0.1%)	3 (1.5%)	1 (0.5%)	1 (0.6%)

^1^ Source: Author’s own calculations based on data from the Polish Central Statistical Office (GUS).

## Data Availability

We analysed official data from the Polish Central Statistical Office.

## Supplementary Materials

The following are available online at https://www.mdpi.com/article/10.3390/ijerph18189921/s1, Table S1: Ratio of deaths caused by suicide in adults aged 65 or older per 100,000 people in European countries.
