# Supplementary material for: Suicide Risk Factors among Polish Adults Aged 65 or Older in 2000–2018 Compared with Selected Countries Worldwide
Source: Int J Environ Res Public Health. 2021 Sep 21;18(18):9921. doi: 10.3390/ijerph18189921 (PMC8465742; doi:10.3390/ijerph18189921)
Supplement: Supplementary file 1 [file ijerph-18-09921-s001.zip › ijerph-1227718-supplementary.pdf]

Table S1. Deaths caused by suicide in adults aged 65 or older per 100,000 people in European countries, and the change in the rates over the period 2017–2000.

Country/Year	Males			Females			Males	Females 2017–2000
	2000	2010	2017	2000	2010	2017	2017–2000	
**United Kingdom**	11.7	9.8	10.1	3.8	3.0	3.2	−14.2%	−16.0%
**Ireland**	11.5	9.5	11.9	4.1	3.2	2.6	3.9%	−37.0%
**Greece**	8.8	9.3	12.3	2.1	1.2	2.6	40.9%	24.0%
**Italy**	24.1	20.4	18.5	6.5	4.3	4.0	−23.4%	−37.7%
**Netherlands**	20.0	16.8	18.5	8.5	6.4	8.9	−7.4%	5.3%
**Norway**	22.5	23.5	19.9	6.3	5.6	4.9	−11.6%	−22.4%
**Slovakia**	40.0	28.7	20.1	8.4	4.3	2.8	−49.6%	−66.5%
**Spain**	31.8	23.3	23.3	8.5	4.9	6.1	−26.8%	−27.7%
**Poland**	31.6	36.1	26.2	6.7	6.1	4.1	−17.0%	−39.6%
**Romania**	30.6	29.8	26.8	8.7	7.8	5.4	−12.2%	−38.5%
**Finland**	37.2	31.2	27.0	10.3	8.2	7.7	−27.6%	−25.4%
**Sweden**	36.1	27.7	28.3	10.2	8.5	8.2	−21.6%	−19.7%
**Czech Republic**	52.3	37.8	32.8	14.0	7.2	8.1	−37.3%	−42.0%
**Germany**	42.4	34.1	35.0	14.1	11.0	9.0	−17.4%	−36.3%
**Portugal**	27.9	45.2	35.0	5.3	9.7	8.0	25.4%	51.2%
**Switzerland**	60.3	36.5	38.2	21.0	9.7	8.4	−36.6%	−60.2%
**Bulgaria**	56.8	40.3	38.4	24.1	12.7	9.7	−32.4%	−59.9%
**Latvia**	90.6	50.6	43.2	24.5	10.0	13.8	−52.3%	−43.6%
**Estonia**	54.5	46.7	46.2	25.0	12.2	11.9	−15.1%	−52.3%
**Austria**	68.7	50.1	56.4	20.6	12.6	10.8	−17.9%	−47.7%
**Hungary**	97.9	80.5	59.4	37.2	19.5	13.7	−39.4%	−63.2%
**Lithuania**	92.3	68.7	68.1	25.9	16.1	16.4	−26.3%	−36.5%
**Slovenia**	106.4	69.8	71.1	28.6	12.2	15.0	−33.2%	−47.7%

Source: Eurostat database.
